# Long-term stress induced cortisol downregulation, growth reduction and cardiac remodeling in Atlantic salmon

**DOI:** 10.1242/jeb.246504

**Published:** 2023-11-16

**Authors:** April Grace R. Opinion, Marine Vanhomwegen, Gudrun De Boeck, Johan Aerts

**Affiliations:** ^1^ University of Antwerp, Department of Biology, ECOSPHERE, 2020 Antwerp, Belgium; ^2^ Ghent University, Department of Biology, Stress Physiology Research Group (StressChron), 8400 Ostend, Belgium; ^3^ Flanders Research Institute for Agriculture, Fisheries and Food, Animal Sciences Unit, Stress Physiology Research Group (StressChron), 8400 Ostend, Belgium

**Keywords:** Chronic stress, Heart morphology, HPI axis, Scales, Salmonids

## Abstract

Stress and elevated plasma cortisol in salmonids have been linked with pathological remodeling of the heart and deterioration of fitness and welfare. However, these associations were based on biomarkers that fail to provide a retrospective view of stress. This study is the first whereby the association of long-term stress, using scale cortisol as a chronic stress biomarker, with cardiac morphology and growth performance of wild Atlantic salmon (*Salmo salar*) is made. Growth, heart morphology, plasma and scale cortisol levels, and expression of genes involved in cortisol regulation of the hypothalamic-pituitary–interrenal axis of undisturbed fish (control) were compared with those of fish exposed daily to stress for 8 weeks. Though scale cortisol levels showed a time-dependent accumulation in both groups, plasma and scale cortisol levels of stress group fish were 29.1% and 25.0% lower than those of control fish, respectively. These results correlated with the overall upregulation of stress-axis genes involved in the systemic negative feedback of cortisol, and local feedback via 11β-hydroxysteroid dehydrogenases, glucocorticoid and mineralocorticoid receptors in the stress treatment at the hypothalamus and pituitary level. These lower cortisol levels were, however, counterintuitive in terms of the growth performance as stress group fish grew 33.7% slower than control fish, which probably influenced the 8.4% increase in relative ventricle mass in the stress group. Though compact myocardium area between the treatments was comparable, these parameters showed significant linear correlations with scale cortisol levels, indicating the involvement of chronic stress in cardiac remodeling. These findings underscore the importance of scale cortisol as biomarker when associating chronic stress with long-term processes including cardiac remodeling.

## Teaser

**Summary:** Examination of the association of long-term stress with cardiac remodeling in Atlantic salmon, using scale cortisol as a chronic stress biomarker.

## INTRODUCTION

Throughout their lifetime, wild and farmed salmonids experience stressful episodes with varying duration, intensity, controllability and predictability, influencing the organism's stress response. Broadly, stress can be defined as a state of threatened homeostasis that can be re-established by a series of adaptive responses (Schreck and Tort, 2016). In teleost fish, the stress response is initiated by two neuroendocrine axes: the hypothalamic-sympathetic-chromaffin (HSC) axis, which leads to the rapid production of catecholamines; and the hypothalamic-pituitary–interrenal (HPI) axis which culminates in the release of glucocorticoids (GCs) (Balasch and Tort, 2019; Schreck and Tort, 2016). In the HPI response, corticotropin-releasing hormone (CRH) is released in the hypothalamus and induces the synthesis of pro-opiomelanocortin (POMC), which is eventually processed into adrenocorticotropic hormone (ACTH) in the pituitary (Huising et al., 2004; Sumpter et al., 1986; Wendelaar Bonga, 2011). ACTH received by the interrenal cells activates steroidogenic acute regulatory protein (STAR), which is the rate-limiting factor for the transport of cholesterol across the mitochondrial membrane for GC synthesis (Stocco, 2000; Wendelaar Bonga, 2011).

GCs, primarily cortisol in teleost fish, are a widely accepted biomarker for stress (Sadoul and Geffroy, 2019) as they mediate the allocation of energy to restore pre-stress conditions (Gorissen and Flik, 2016), and are eventually downregulated through negative feedback mechanisms at different levels of the HPI axis (Alderman et al., 2012; Barton, 2002). For instance, cortisol directly exerts negative feedback on CRH synthesis and ACTH secretion (Bernier et al., 2004; Bernier and Peter, 2001; Fryer et al., 1984). Glucocorticoid receptors (GRs) and mineralocorticoid receptors (MRs), which mediate the actions of cortisol by activating or inhibiting the expression of target genes, are also involved in the negative feedback regulation of the HPI axis, primarily at the level of the hypothalamus and pituitary (Bury et al., 2003; Faught and Vijayan, 2018). Furthermore, cortisol can be regulated enzymatically through 11β-hydroxysteroid dehydrogenase 2 (11β-HSD2), which inactivates cortisol to cortisone (Baker, 2004; Chapman et al., 2013).

While the stress response is generally adaptive under mild or short-term stress conditions, failure to regain homeostasis during severe or prolonged stress conditions leads to chronic stress and may subject the individual to the detrimental effects of GC-mediated actions. Moreover, the stress response is an energy-demanding process, and chronic stress renders energy unavailable for important life processes including growth, digestion, immunity and reproduction (Schreck and Tort, 2016). The attempt to rectify this situation is termed ‘allostasis’, wherein physiological and behavioral set points of regulatory mechanisms are adjusted to optimize organismal performance under predicted environmental demands at minimal cost (Schreck, 2010; Schreck and Tort, 2016; Sterling and Eyer, 1988). As such, low allostatic load (or eustress) can improve the performance of the animal, whereas allostatic overload (or distress) encountered during chronic stress can become a pathophysiological condition (Schreck and Tort, 2016). Chronic stress-induced maladaptation is particularly relevant in farmed fish that are confined in systems where stressors such as crowding, handling, infections and sub-optimal water quality are frequently encountered (Balasch and Tort, 2019; Conte, 2004). On top of that, chronic stress in the natural environment is becoming more concerning as a result of anthropogenic activities that disrupt aquatic habitats and exacerbate the stress severity during inherently stressful life processes (i.e. migration and spawning).

Stress has been associated with cardiac remodeling in salmonids. Cardiac growth, for instance, is a long-known adaptive response that enhances myocardial performance and cardiac pumping capacity during stressful periods, including thermal acclimation and sexual maturation of salmonids (Gamperl and Farrell, 2004; Keen et al., 2017; Klaiman et al., 2011). Individual cortisol responsiveness to stress has also been linked with cardiac remodeling including heart growth and thickening of the compact myocardium in rainbow trout (*Oncorhynchus mykiss*) and wild-strain brown trout (*Salmo trutta*) (Johansen et al., 2011), and such remodeling is directly induced by cortisol (Johansen et al., 2017; Norstrud et al., 2018). Nevertheless, the observed cortisol-induced remodeling seems maladaptive as it corresponded with impaired cardiovascular performance and upregulation of cardiac hypertrophy and pathology molecular markers (Johansen et al., 2017). This maladaptation is problematic as cardiac deformities and failure were increasingly associated with devastating mortalities in salmonids (Brocklebank and Raverty, 2002; Grefsrud et al., 2018; Poppe et al., 2007). For instance, cardiac arrest triggered by acute temperature rise caused die-offs of wild salmon in Alaska (MacArthur, 2019). In aquaculture, clear signs of compromised welfare and cardiac abnormalities were reported during massive mortalities that were seemingly prompted by acutely stressful interventions (Brocklebank and Raverty, 2002; Grefsrud et al., 2018; Poppe et al., 2007).

Long-term elevated cortisol, as seen in chronic stress, is proposed as an underlying factor in the maladaptive cardiac response and consequent mortalities in salmonids. However, existing data regarding the association between stress and cardiac morphology of salmonids are based on plasma cortisol levels (Johansen et al., 2011; 2017; Nørstrud et al., 2018), which merely reflect the cortisol status at sampling and fail to provide information on cortisol exposure throughout past life periods (Aerts et al., 2015; Sadoul and Geffroy, 2019; Oliveira et al., 2013). As such, the association of chronic stress with fish performance and cardiac remodeling remains unexplored. Fish scales, in contrast, persistently incorporate cortisol and may therefore provide a view of stress levels experienced by fish over time, making it a promising biomarker for chronic stress (Aerts et al., 2015; Laberge et al., 2019).

This study aimed to be the first to establish the association of chronic stress, quantified by scale cortisol, with cardiac morphology and growth performance using wild-strain *Salmo salar* juveniles as experimental species. *Salmo salar* is an important species for aquaculture, restocking and restoration efforts. In these activities, juveniles tend to be more exposed to stress (i.e. handling, crowding and transportation) as these individuals are released into the wild or introduced into grow-out aquaculture facilities as smolts. For restoration efforts, juveniles are often of wild or undomesticated parentage, which may respond differently to stressors than their domesticated counterparts, given the reported attenuation of the cortisol response induced by domestication (Lu et al., 2022; Milla et al., 2021). Taken together, wild-strain juveniles appear to be at higher risk of chronic stress and cortisol-mediated effects. Here, growth, heart morphology, cortisol levels in plasma and scales, and expression of key genes involved in the cortisol regulation of the HPI axis of undisturbed (control) fish were compared with fish exposed daily to unpredictable chronic stress for 8 weeks. We hypothesized that exposure to unpredictable chronic stress (UCS) would induce long-term endogenous upregulation of cortisol in wild-strain *S. salar*, quantified by scale cortisol. Consequently, this cortisol upregulation will induce cardiac remodeling and impair organismal performance.

## MATERIALS AND METHODS

### Acclimatization and experimental design

All experimental procedures complied with the Federation of European Laboratory Animal Science Associations' regulations and were approved by the University of Antwerp's ethics committee (permit number: 2020-67). *Salmo salar* juveniles (31.87±1.3 g, mean±s.e.m.), which are first-generation fish (male and female) from wild-caught parents, were obtained from SPW Agriculture, Ressources Naturelles et Environnement (Liege, Belgium), and transported to the mesocosm research facilities of the University of Antwerp, where they were acclimated to laboratory conditions for 4 months (February to May 2022) prior to the experiment. The fish were distributed and maintained in four circular tanks (3.76 m^3^ volume, 2 m diameter, 1.2 m height; filled with 3.10 m^3^ recirculated freshwater) at a stocking density of 33 fish per tank (0.3 g l^−1^). Each tank was equipped with an EconoBead Complete Filtration system (AquaForte, Verghel, The Netherlands) composed of a 300 µm stainless sieve (Midi Sieve XL 300micron), bead filter (EconoBead-60) and UV filter (75 W, Midi Power UV-C T5). Air lines and thermoregulators (TK-9000, Teco, Ravenna, Italy) were installed in each tank. Water temperature was increased from 6 to 13°C at a rate of 1°C every week, and was maintained at 13°C for 21 days before the experiment. The fish were fed automatically (Fish Feeder Easy, Velda, Enschede, The Netherlands) with commercial feed (Crystal 2 mm, Alltech Coppens, Helmond, The Netherlands) 3 times a day (08:00 h, 14:00 h and 20:00 h) at a 3% feeding rate. Passive Integrated Transponders (ID-100C, injected using an IM-300C Pistol Grip Implanter, Trovan, Yorkshire, UK) were implanted in fish at the right dorsolateral muscle area 40 days before the start of the experiment.

At the onset of the experiment, fish (61.32±0.82 g, mean±s.e.m.) were exposed to either of two treatments: (1) control, where fish were left undisturbed; and (2) stress, where fish were subjected daily to a UCS protocol using stimuli that are commonly experienced in aquaculture. The four tanks where the fish were acclimated were randomly assigned to one of the two treatments using a random number generator (random.org) producing two replicate tanks for each treatment. The UCS protocol of the stress treatment involved application of acute stressors once per day, where the type, duration and timing of stressors used were randomized throughout the experiment (Tables S1 and S2) including 5–10 min chasing, 3–6 min crowding, 3–7 min netting with 5–30 s air exposure, and temperature shock (up to 3°C increase in temperature). The experiment ran from May to July 2022.

Throughout the acclimation and experimental period, fish were subjected to a natural light regime. Temperature and dissolved oxygen (DO) were recorded daily by a portable meter (WTW Profiline 3310 with CellOx 325 probe, Xylem, DC, USA), and were maintained below 13°C and above 85%, respectively. Ammonia, nitrite and nitrate were measured by Tetra test kits daily (Blacksburg, VA, USA), and were maintained below 0.25 mg NH_3_/NH_4_^+^ l^−1^, 0.3 mg NO_2_^−^ l^−1^ and 25 mg NO_3_^−^ l^−1^, respectively, by partial (20%) daily water exchange.

### Sample collection

Sample collection was done at 4 time points (week 0, 2, 5 and 8). On the sampling days, no stressor was applied and the sample collection was conducted around 09:00–12:00 h. For each time point, 6 fasted fish (24 h) from each tank were netted, and euthanized by an overdose of tricaine methanesulfonate (1 g l^−1^, MS-222, Acros Organics, Geel, Belgium). The fish tags were recorded (LID-560ISO Pocket reader, Trovan RFID Systems Ltd, Melton, UK) and body mass (BM) and length were registered (SI-203, Denver Instrument, Bohemia, NY, USA). Blood samples were immediately collected by puncturing the caudal vein with a heparinized (1000 IU ml^−1^, heparin lithium salts from porcine mucosa, Sigma-Aldrich, St Louis, MO, USA) tuberculin syringe fitted with a 23-G needle (Terumo, Leuven, Belgium), and were spun (7 min at 9300 rpm at 4°C, 5415R microcentrifuge, Eppendorf, Hamburg, Germany) to obtain plasma. Ontogenetic scales were collected from the left dorsolateral area (between the operculum and first dorsal spine). Fish were then decapitated, and the hypothalamus, pituitary and head kidney were dissected and preserved in RNAlater (ABP Bioscience, Rockville, MD, USA) for gene expression analysis. Thereafter, heart samples were excised, and the atrium and bulbus arteriosus were carefully removed before the ventricles were blotted dry and weighed (SI-203, Denver Instrument). At week 8, the ventricles were fixed in 10% neutral buffered formaldehyde (Sigma-Aldrich) for histological analysis. Plasma and scales samples were frozen and stored at −20°C for subsequent cortisol analysis. Tissue samples for gene expression and histology analysis were incubated at room temperature overnight, and stored at −20°C and 4°C until analysis, respectively. During the exposure, sampling and sample analyses, the investigators were not blinded to the treatment.

Individual specific growth rate [SGR, %BM day^−1^; 100×(lnBM_f_−lnBM_i_)/*t*, where BM_f_ and BM_i_ represent the final and initial BM in grams, respectively, and *t* is the growth period in days) were calculated. The relative ventricle mass (RVM) was determined by dividing the ventricle mass by fish BM (g g^−1^×100).

### Scale cortisol analysis

Scale cortisol was analyzed using ultra-performance liquid chromatography–tandem mass spectrometry (UPLC-MS/MS) as described in Aerts et al. (2015). Defrosted scale samples were carefully wiped with water-moistened paper tissue to remove the mucus. UPLC-MS/MS analysis for scale cortisol included exogenous GCs commonly encountered in the water and in the mucus such as tertrahydrocortisol and tetrahydrocortisone to ensure no contamination by external GCs or GCs from the hands of personnel handling the scales. The air-dried scales were then weighed (XP205, Mettler-Toledo, Greifensee, Switzerland) and transferred to PowerBead tubes (Ceramic 2.8 mm, Qiagen, Hilden, Germany). The samples were then homogenized in a PowerLyzer 24 (3500 rpm, 3 times 10 s with 15 s dwell time; Qiagen) to homogenize. Homogenized samples were quantitively transferred with 8 ml of methanol into 12 ml glass tubes to which 10 μl cortisol-d_4_ (0.5 ng μl^−1^, CDN Isotopes, Pointe-Claire, QC, Canada) was added as internal standard. The samples were vortexed (Genie 2, Scientific Industries, NY, USA) for 30 s, placed in an overhead shaker (Multi RS-60, Biosan, Riga, Latvia) at 60 rpm for 1 h at room temperature, and centrifuged (5810-R, Eppendorf) for 10 min at 3500 ***g*** at 7°C. All supernatant was transferred into a new 12 ml glass tube, evaporated to dryness at 60°C by a nitrogen evaporator (TurboVap^®^ Classic LV, Biotage, Uppsala, Sweden), and reconstituted in 5 ml H_2_O/methanol (80/20 v/v).

After conditioning the C_18_ solid-phase extraction columns (C_18_-Max, 500 mg, 6 ml, S*Pure, Singapore) with 3 ml methanol followed by 3 ml Type-I HPLC-grade water, the prepared samples were loaded. The columns were washed with 4.5 ml H_2_O/methanol (65/35 v/v) and the retained compounds were eluted with 2.5 ml H_2_O/methanol (20/80 v/v) into 12 ml glass tubes, then evaporated to dryness at 60°C by a nitrogen evaporator. The samples were finally reconstituted in 50 μl H_2_O/methanol (80/20 v/v) in vials with inserts and analyzed on an Acquity UPLC BEH C18 (1.7 µm; 2.1 mm and 100 mm) column using UPLC-MS/MS (Xevo TQS, Waters, Milford, MA, USA).

A set of calibration standards, ranging from 0.1 to 5 ng g^−1^, was prepared by adding 10 µl of 0.5 ng µl^−1^ cortisol-d_4_ solution to 0.1, 0.5, 1.0, 2.5 and 5.0 µl of 0.1 ng µl^−1^ standard mix solution, and diluting these to generate 100 µl of H_2_O/methanol (20/80 v/v) solution. Blank (100 µl H_2_O/methanol, 20/80 v/v) and positive controls (2.5 µl of 0.1 ng µl^−1^ standard mix and 10 µl of 0.5 ng l^−1^ cortisol-d_4_ in 100 µl H_2_O/methanol, 20/80 v/v) were prepared for quality checking. Data analysis was performed using Quanlynx software (Waters); analysis results were reported as the value (μg kg^−1^) ±the expanded measurement uncertainty (μg kg^−1^) with a coverage factor (*k*) of 2 (95% confidence interval).

### Plasma analysis

UPLC-MS/MS was also used to quantify the cortisol level in plasma. To prepare the plasma samples, 10 µl of defrosted plasma was added to 4989 µl Type-I HPLC-grade water. Subsequently, 1 µl of 0.05 ng µl^−1^ cortisol-d_4_ was added as an internal standard, and the samples were vortexed for 30 s to homogenize, then purified by solid-phase extraction and analyzed by UPLC-MS/MS, following the same protocol used for scale cortisol. A similar quality assessment method was also employed. However, the calibration standards for cortisol analysis of plasma ranged from 1 to 50 ng ml^−1^. This calibration set was prepared by mixing 10 µl of 0.5 ng µl^−1^ cortisol-d_4_ solution with 1, 5, 10, 25 and 50 µl of 0.1 ng µl^−1^ standard mix solution in 100 µl of H_2_O/methanol (20/80 v/v) solution. Results were reported as the value (µg l^−1^) ±the expanded measurement uncertainty (μg l^−1^) with a coverage factor (*k*) of 2 (95% confidence interval).

### Gene expression analysis

The expression levels of genes involved in cortisol regulation were quantified at relevant levels of the HPI axis including: corticotropin releasing hormone (*crh*), proopiomelanocortin 1 (*pomca1*), proopiomelanocortin 2 (*pomca2*), proopiomelanocortin b (*pomcb*), steroidogenic acute regulatory protein (*star*), glucococorticoid receptor (*gr*), mineralocorticoid receptor (*mr*) and 11β-hydroxysteroid dehydrogenase 2 (*11β-hsd2*). To assess the relative expression of target genes, RNA was isolated from tissues using RNeasy Plus 96 kit (Qiagen) following the manufacturer's manual. Tissue samples in PowerBead tubes were homogenized in a PowerLyzer24 (3500 rpm, 3 times 45 s with 30 s dwell time between cycles). The quality of RNA extracts was checked through the 280/260 nm and 260/230 nm absorbance ratios determined by QIAxpert (Qiagen), and gel electrophoresis was performed to evaluate RNA integrity. The concentration of extracted RNA was also measured by QIAxpert at 260 nm and was standardized to 30 ng µl^−1^ in all samples. iScript cDNA synthesis kit (Bio-Rad Laboratories, Hercules, CA, USA) was used for cDNA synthesis where 5 µl of iScript master mix, composed of 1 part reverse transcriptase and 4 parts reaction mix, was added to 15 µl of standardized RNA extracts. No-template controls and no-reverse transcriptase controls were included for quality assessment. Reverse transcription was performed at 46°C for 20 min and 95°C for 1 min (Mastercycler X50 s, Eppendorf), and the generated cDNA samples were diluted to 2.5 ng µl^−1^. The reaction setup for real-time PCR (qPCR) included 2 µl of 2.5 ng µl^−1^ cDNA sample as template, 0.25 µl of 5 µmol l^−1^ each forward and reverse primer pair, and 2.5 µl of SsoAdvanced Universal SYBR Green Supermix (Bio-Rad Laboratories). The reaction mix was loaded into a 384-well plate (hard-shell PCR plates, thin-wall, Bio-Rad Laboratories) and the run was performed in a CFX-384 Touch Real-Time PCR Detection System (Bio-Rad Laboratories) with reaction conditions of 95°C for 30 s followed by 40 cycles of 95°C for 10 s and 60°C for 20 s. No-template controls were included for quality assessment, and melt-curve analyses were done to check the assay specificity. The target gene mean normalized expression was determined using a normalization factor calculated by qBase+ software (CellCarta, Montreal, QC, Canada), based on three housekeeping genes: ribosomal protein S20 (*s20*), elongation factor 1α (*elf1a*) and beta-actin (*β-actin*).

### Histology

Heart samples at week 8 were rinsed three times for 10 min each in 0.01 mol l^−1^ PBS (pH 7.4) and stored in 0.01 mol l^−1^ PBS (pH 7.4) containing 0.1% sodium azide at 4°C until further handling. After processing in an STP120 spin tissue processor (Epredia, Machelen, Belgium), hearts were embedded in paraffin after which 5 µm transverse sections were stained with hematoxylin & eosin (HE) and scanned with a Zeiss Axio Scan.Z1 slide scanner (Carl Zeiss, Oberkochen, Germany) at ×10 magnification. The transverse sections were taken from the same height of each heart: 1800 µm from the apex of the ventricle. QuPath software (Bankhead et al., 2017) was used to analyze the tissue sections. The spongy myocardium was first manually delineated in every section. Next, the overall tissue was automatically detected in the image based on a user-defined fixed threshold. The compact myocardium was then identified as the area between the whole tissue and the manually delineated spongy myocardium. The average width of the compact myocardium was quantified based on the Euclidean distance map. The width of the compact myocardium was measured for every point along the middle of the compact myocardium (see Results, ‘Heart morphology’). From all these measurements, the average width was reported for every tissue section as the absolute distance in µm. Finally, the extra-bundular sinus was detected based on a user-defined threshold combined with a minimal area filter.

### Statistical analysis

The sample size used in the study was determined based on power analyses (G*Power version 3.1) with statistical power of 95% and a type I error rate of 0.05 considering scale cortisol data by Laberge et al. (2019). Data analysis was performed in R (version 4.2.2; R studio version 2022 12.0+353). Linear mixed models (lmer) were run to determine the main and interactive effects of treatment (two levels, fixed factor) and time points (three to four levels, fixed factor) on plasma and scale cortisol levels, expression of genes involved in cortisol synthesis (*crh*, *pomca1*, *pomca2*, *pomcb* and *star*) and regulation (*gr*, *mr* and *11β-hsd2* at all levels of the HPI axis), growth rate and RVM. To determine the effect of treatment (two levels, fixed factor) on the ventricular morphology (average compact myocardium width, compact/spongy myocardium area ratio and relative extra-bundular sinus area), lmer models were also run. Tank ID was included as a random effect in all models, and the assumptions of the models were checked. Scale cortisol, *crh*, *star* and *11β-hsd2* (in head kidney) datasets were square-root transformed while *pomca1*, *pomca2*, *pomcb*, *mr* (in hypothalamus) and *11β-hsd2* (in hypothalamus) were log transformed to fit the assumption of normality. Tukey's *post hoc* tests (Tukey-adjusted least square means) were run to determine statistical differences among treatment groups and fixed factor levels. The associations of ventricle morphological parameters with plasma and scale cortisol were assessed by simple linear regression (lm) with Pearson's correlation coefficient as a measure of the linear relationship. Statistical significance was accepted at a probability level less than or equal to 0.05 (*P*≤0.05). Data are presented as means±s.e.m.

## RESULTS

### Cortisol level

**Fig. 1.**
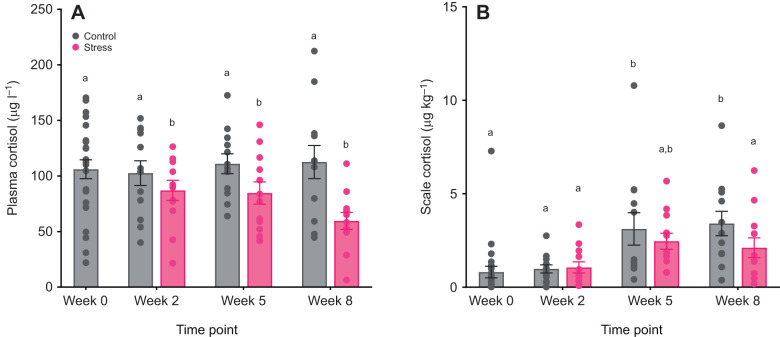
**Plasma and scale cortisol levels of control and stressed Atlantic salmon (*Salmo salar*).** Fish were undisturbed (control) or exposed to unpredictable chronic stress, and plasma (A) and scale (B) cortisol were measured at different sampling points (week 0, 2, 5 and 8). Bar graphs show means±s.e.m.; circles represent individual data points (week 0, baseline control: *n*=24; weeks 2–8: *n*=12 per treatment per time point). Statistical significance was determined by linear mixed models with Tukey-adjusted least square means for multiple comparisons. Different lowercase letters indicate statistical differences (*P*<0.05) among treatments and time points.

Time had a slight but significant effect on plasma cortisol levels (*F*_3,86_=2.90, *P*<0.05), which was mainly driven by the 25% decrease of plasma cortisol levels over time in fish exposed to the stress treatment (*F*_1,70_=31.69, *P*<0.001; Fig. 1A). Cortisol level in scales showed a time-dependent accumulation (*F*_3,84_=20.03, *P*<0.001) in both treatments, but the degree of accumulation was significantly different between treatments (*F*_1,84_=12.84, *P*<0.001), where fish in the stress treatment accumulated 29% lower scale cortisol compared with control (Fig. 1B). The interaction between time and treatment was not significant in both scale (*F*_2,85_=0.99, *P*=0.38) and plasma cortisol (*F*_2,85_=1.88, *P*=0.15).

### Cortisol regulation

**Fig. 2.**
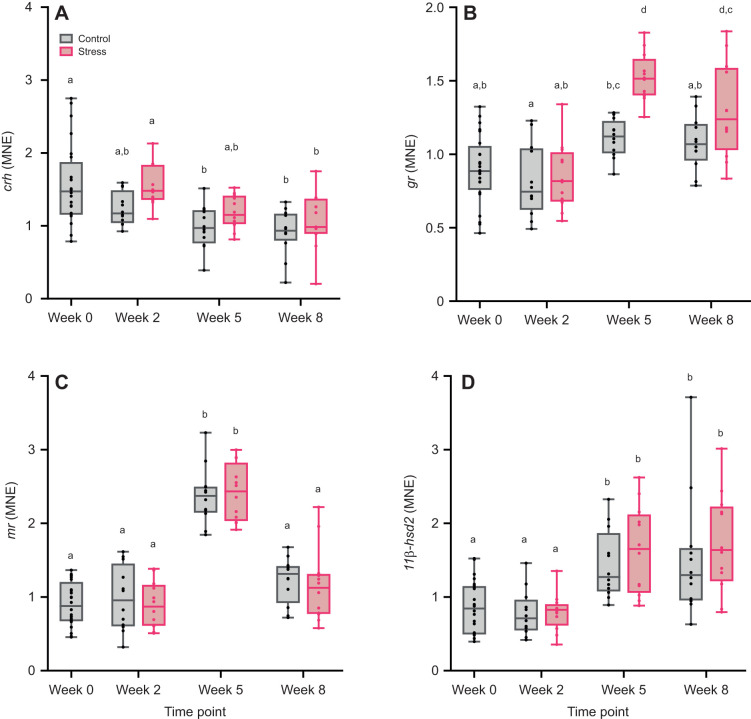
**Expression of genes involved in cortisol regulation in the hypothalamus of control and stress-exposed Atlantic salmon.** Mean normalized expression (MNE) of corticotropin releasing hormone (*crh*, A), glucocorticoid receptor (*gr*, B), mineralocorticoid receptor (*mr*, C) and 11β-hydroxysteroid dehydrogenase 2 (*11β-hsd2*, D) was measured at different sampling points (week 0, 2, 5 and 8). Boxplots show medians, upper and lower quartiles, and maximum and minimum (whiskers); circles represent individual data points (week 0, baseline control: *n*=24; weeks 2–8: *n*=11–12 per treatment per time point). Statistical significance was determined by linear mixed models with Tukey-adjusted least square means for multiple comparisons. Different lowercase letters indicate statistical differences (*P*<0.05) among treatments and time points.

The expression of genes involved in *de novo* cortisol synthesis (*crh*, *pomca1*, *pomca2*, *pomcb* and *star*) and cortisol regulation (*gr*, *mr* and *11β-hsd2*) was quantified at relevant levels of the HPI axis. In the hypothalamus, both time (*F*_3,88_=14.24, *P*<0.001) and treatment (*F*_1,88_=6.22, *P*<0.05) had significant effects on *crh* expression but the interaction effect was not statistically significant (*F*_2,88_=0.26, *P*=0.77; Fig. 2A). Expression of *crh* was generally higher in stressed fish compared with control, but it was decreasingly expressed in both treatments over time. As for the genes involved in cortisol regulation, *gr* was significantly affected by time (*F*_3,78_=23.11, *P*<0.001), treatment (*F*_1,23_=12.71, *P*<0.001) and their interaction (*F*_2,86_=4.98, *P*<0.001; Fig. 2B). *Post hoc* tests indicated that *gr* was generally upregulated at weeks 5 and 8, but stress treatment induced higher upregulation compared with control. *mr* expression was only affected by time (*F*_3,89_=46.73, *P*<0.001) but not by treatment (*F*_1,89_=0.27, *P*=0.60) and interaction of factors (*F*_2,89_=0.18, *P*=0.83), which was manifested by the proportional upregulation of *mr* in the treatments at week 5 (Fig. 2C). Time had a significant effect on *11β-hsd2* expression (*F*_3,89_=19.22, *P*<0.001), but treatment (*F*_1,89_=1.21, *P*=0.27) and its interaction with time point (*F*_2,89_=0.17, *P*=0.85) did not affect this gene (Fig. 2D). *Post hoc* tests showed a comparable increase in the expression level of *11β-hsd2* in both treatments at weeks 5 and 8 compared with other time points.

**Fig. 3.**
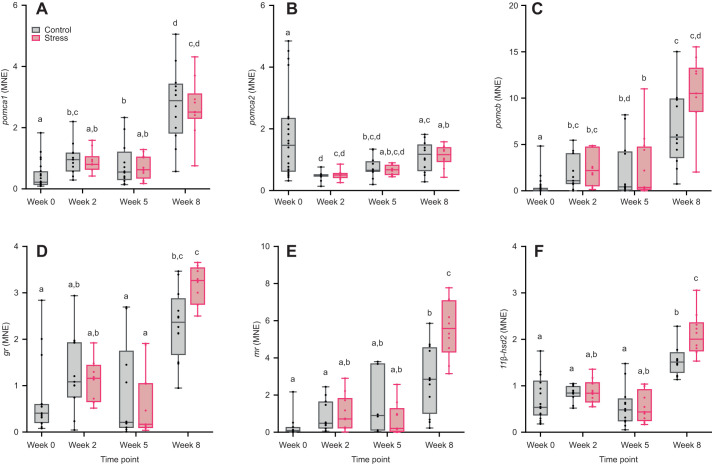
**Expression of genes involved in cortisol regulation in the pituitary of control and stress-exposed Atlantic salmon.** MNE of proopiomelanocortin 1 (*pomca1*, A), proopiomelanocortin 2 (*pomca2*, B), proopiomelanocortin b (*pomcb*, C), *gr* (D), *mr* (E) and *11β-hsd2* (F) was measured at different sampling points (week 0, 2, 5 and 8). Boxplots show medians, upper and lower quartiles, and maximum and minimum (whiskers); circles represent individual data points (week 0, baseline control: *n*=11–24; weeks 2–8: *n*=8–12 per treatment per time point). Statistical significance was determined by linear mixed models with Tukey-adjusted least square means for multiple comparisons. Different lowercase letters indicate statistical differences (*P*<0.05) among treatments and time points.

In the pituitary, time significantly affected *pomca1* and *pomcb* expression (*pomca1*: *F*_3,75_=36.69, *P*<0.001; *pomcb*: *F*_3,76_=26.23, *P*<0.001), but treatment (*pomca1*: *F*_2,27_=3.07, *P*=0.09; *pomcb*: *F*_2,31_=1.97, *P*=0.17) and interaction effects were not significant (*pomca1*: *F*_2,80_=0.03, *P*=0.97; *pomcb*: *F*_2,80_=0.03, *P*=0.97; Fig. 3A,C). Both *pomca1* and *pomcb* were increasingly expressed in the treatments over time, with a peak at week 8. *pomca2* expression was affected by time (*F*_3,71_=16.73, *P*<0.001) and treatment (*F*_1,19_=4.57, *P*<0.05) but not by their interaction (*F*_2,79_=0.08, *P*=0.92; Fig. 3B), where it was significantly downregulated at week 2 and increased to baseline levels at week 8 in both treatments. Though the treatment effect was not significant (*F*_1,3_=0.27, *P*=0.64), time (*F*_3,36_=31.93, *P*<0.001) and its interaction with treatment (*F*_2,69_=3.54, *P*<0.05) significantly affected the expression level of *gr* (Fig. 3D). The expression of *mr* was affected by time (*F*_3,23_=33.78, *P*<0.001) but the effect of treatment (*F*_1,4_=1.76, *P*=0.25) was not significant (Fig. 3E). However, there was a significant interaction effect between treatment and time point (*F*_2,60_=10.23, *P*<0.001), where *mr* was upregulated at week 8 and the upregulation was higher in the stress treatment compared with control. Treatment (*F*_1,11_=7.19, *P*<0.05), time (*F*_3,59_=36.98, *P*<0.001) and their interaction (*F*_2,74_=3.13, *P*<0.05) significantly affected the expression of *11β-hsd2* (Fig. 3F). According to the *post hoc* test, *11β-hsd2* was significantly upregulated at week 8 but the expression was higher in stress compared with control treatments.

**Fig. 4.**
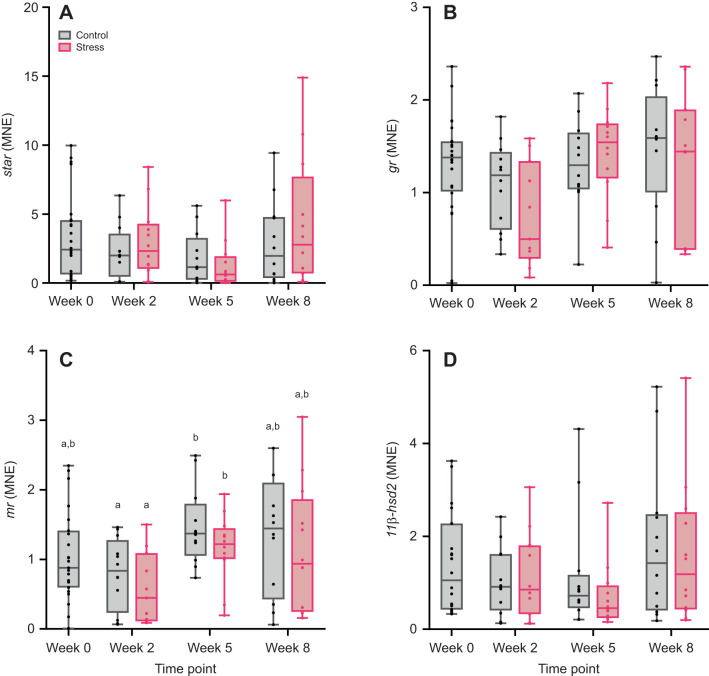
**Expression of genes involved in cortisol regulation in the head kidney of control and stress-exposed Atlantic salmon.** MNE of steroidogenic acute regulatory protein (*star*, A), *gr* (B), *mr* (C) and *11β-hsd2* (D) was measured at different sampling points (week 0, 2, 5 and 8). Boxplots show medians, upper and lower quartiles, and maximum and minimum (whiskers); circles represent individual data points (week 0, baseline control: *n*=22–24; weeks 2–8: *n*=11–12 per treatment per time point). Statistical significance was determined by linear mixed models with Tukey-adjusted least square means for multiple comparisons. Different lowercase letters indicate statistical differences (*P*<0.05) among treatments and time points.

Expression of *star* in the head kidney was not affected by treatment (*F*_1,3_=0.20, *P*=0.71), time (*F*_3,69_=2.41, *P*=0.07) or their interaction (*F*_2,86_=0.76, *P*=0.47; Fig. 4A). Treatment (*F*_1,87_=0.56, *P*=0.45), time (*F*_3,87_=1.76, *P*=0.16) and their interaction (*F*_2,87_=0.32, *P*=0.72) did not significantly affect the expression of *11β-hsd2* in the head kidney (Fig. 4D). There was a significant effect of time on *gr* and *mr* expression (*gr*: *F*_3,71_=3.41, *P*<0.05; *mr*: *F*_3,69_=4.76, *P*<0.01), but treatment (*gr*: *F*_1,11_=0.88, *P*=0.37; *mr*: *F*_1,12_=1.52, *P*=0.24) and interaction effects (*gr*: *F*_2,84_=1.00, *P*=0.37; *mr*: *F*_2,85_=0.07, *P*=0.93) were not significant (Fig. 4B,C). Nevertheless, *post hoc* tests showed no significant differences in the expression of *gr* among treatment–time point combinations, while the expression of *mr* was significantly lower at week 2 than at week 5 in both treatments.

### Growth

**Fig. 5.**
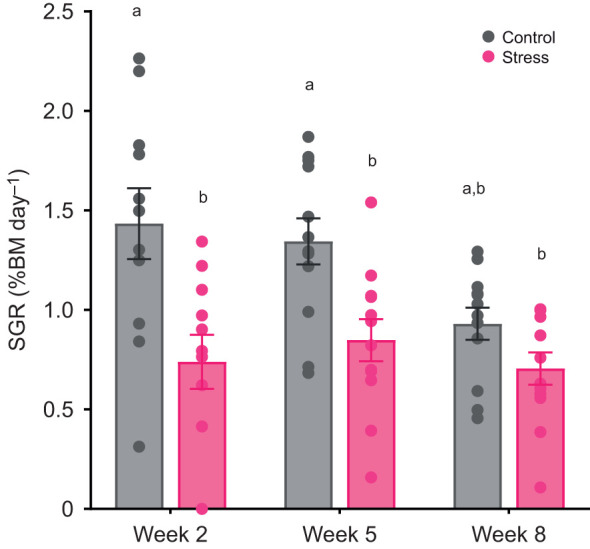
**Specific growth rate of control and stress-exposed Atlantic salmon.** Specific growth rate [SGR; percentage body mass (BM) per day] was measured at different sampling points (week 0, 2, 5 and 8). Bar graphs show means±s.e.m.; circles represent individual data points (*n*=11–12 per treatment per time point). Statistical significance was determined by linear mixed models with Tukey-adjusted least square means for multiple comparisons. Different lowercase letters indicate statistical differences (*P*<0.05) among treatments and time points.

Overall, stress treatment caused a significant 33.7% reduction in growth rate relative to the control (*F*_1,66_=14.76, *P*<0.001), but the effect of time (*F*_2,66_=2.90, *P*=0.06) did not reach statistical significance (Fig. 5). The interaction effect of time and treatment on growth rate was not significant (*F*_2,66_=0.73, *P*=0.49).

### Heart morphology

**Fig. 6.**
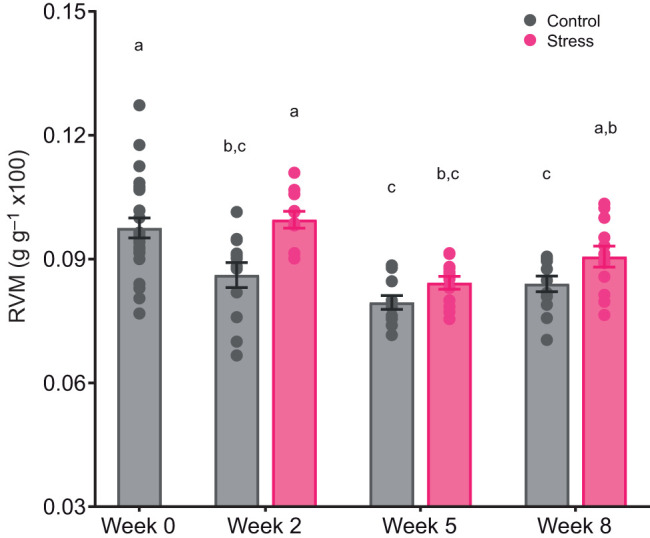
**Relative ventricular mass of control and stress-exposed Atlantic salmon.** Relative ventricular mass (RVM; ventricle mass divided by fish BM) was measured at different sampling points (week 0, 2, 5 and 8). Bar graphs show means±s.e.m.; circles represent individual data points (week 0, baseline control: *n*=24; week 2–8: *n*=12 per treatment per time point). Statistical significance was determined by linear mixed models with Tukey-adjusted least square means for multiple comparisons. Different lowercase letters indicate statistical differences (*P*<0.05) among treatments and time points.

**Fig. 7.**
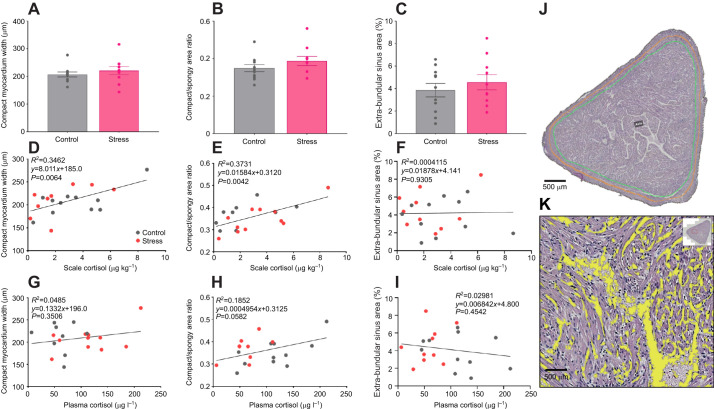
**Ventricular morphology of control and stress-exposed Atlantic salmon.** (A–C) Bar graphs show mean±s.e.m. compact myocardium width (A), compact/spongy myocardium area ratio (B) and relative extra-bundular sinus area (C) at week 8; circles represent individual data points (*n*=10–11 per treatment). Statistical significance (*P*≤0.05) between treatments was determined by linear mixed models with Tukey *post hoc* test. (D–I) Each histological parameter is plotted against scale cortisol (D–F) and plasma cortisol (G–I) for control and stressed fish. (J,K) Representative image of hematoxylin and eosin-stained ventricle showing annotated areas of compact and spongy myocardium (J: black line, ventricle outer bound; green line, border between compact outer region and spongy myocardium inner region; and orange line, middle of the compact myocardium for width calculation), and extra-bundular sinus area (K, yellow areas; boxed region in the inset indicates the location in J). The association of heart morphological parameters with cortisol was assessed by simple linear regression with Pearson's correlation (*R*^2^) coefficient as a measure of the linear relationship.

Treatment (*F*_1,40_=22.26, *P*<0.001) and time (*F*_3,81_=22.60, *P*<0.001) had significant effects on RVM (Fig. 6). Stressed fish generally exhibited 8.4% higher RVM compared with control, and the RVM of control seemed to decrease with time. The interaction between treatment and time was not significant (*F*_2,83_=1.77, *P*=0.18). Histological results on the compact myocardium width (*F*_1,19_=0.67, *P*=0.42; Fig. 7A), compact/spongy myocardium ratio (*F*_1,19_=1.56, *P*=0.23; Fig. 7B) and extra-bundular sinus area (*F*_1,2_=0.22, *P*=0.68; Fig. 7C) between stressed and control fish were comparable at week 8. The correlations of average compact myocardium width (*R*^2^=0.35, *P*<0.01; Fig. 7D) and compact/spongy myocardium ratio (*R*^2^=0.37, *P*<0.01; Fig. 7E) were significantly linear when plotted against scale cortisol but not against plasma cortisol (width: *R*^2^=0.04, *P*=0.35; Fig. 7G; ratio: *R*^2^=0.19, *P*=0.06; Fig. 7H). The correlation of extra-bundular sinus was not significantly linear with scale (*R*^2^<0.001, *P*=0.93; Fig. 7F) or plasma cortisol (*R*^2^=0.03, *P*=0.45; Fig. 7I).

## DISCUSSION

Long-term upregulation of cortisol has been considered as an underlying factor in the maladaptive effects of chronic stress on fish performance. Nevertheless, existing associations of cortisol and fish performance are based on bio-indicators which do not provide a retrospective view of the cortisol response. For the first time, the temporal profile of scale cortisol, as a potential chronic stress biomarker, in wild-strain *S. salar* exposed to UCS was determined, and its association with cardiac morphology and growth was established. Our novel results further support the suitability and applicability of scale cortisol to quantify chronic cortisol levels as indicated by the time-dependent accumulation of cortisol in scales proportional to the plasma levels. Furthermore, UCS induced a downregulation of the cortisol response, which correlated with the changes in stress axis gene expression. Though there was a lack of concordance between cortisol and UCS-induced growth reduction, scale cortisol showed significant linear correlation with compact myocardium width and area suggesting the involvement of cortisol in cardiac remodeling.

### Stress response to UCS

Though stable throughout the experiment, the observed plasma cortisol level of the control group was relatively higher than the reported levels in stress exposure studies on *S. salar* juveniles (Lai et al., 2021; Madaro et al., 2015; 2016; Pankhurst et al., 2008). The interstudy differences in the control cortisol levels can be attributed to the source and life history of the *S. salar* used. Unlike the wild strain investigated in this experiment, other experiments used domesticated fish that may have an attenuated stress response through domestication (Lu et al., 2022; Palińska-Żarska et al., 2021). There is growing evidence that the longer fish are domesticated, the lower their cortisol levels are relative to wild fish following stress exposure (Lu et al., 2022; Milla et al., 2021). Moreover, the undisturbed cortisol levels in domesticated salmonids seemed lower than those in their wild counterparts (Lepage et al., 2000; Mazur and Iwama, 1993) which was probably due to uncontrollable stress stimuli from the rearing environment (Madliger and Love, 2014) to which the wild-strain fish are less adapted. Indeed, the cortisol levels of the control observed in this study are comparable with reported levels in wild-strain *S. salar* of similar size (Fjelldal et al., 2020)*.* However, it is also important to note that the gradual increase in temperature during acclimation (February to April) coupled with the longer daylength when the experiment was conducted (May to July) may have induced smoltification, and consequently influenced cortisol levels. Being one of the major endocrine regulators of the osmoregulatory process, cortisol tends to increase during smoltification, which normally occurs around spring, triggered by photoperiod and temperature cues (Culbert et al., 2022; McCormick et al., 2007; McCormick, 2012). Of note, the analytical methods used to determine cortisol levels are different in previous studies, which necessitates caution when comparing interstudy results (Aerts, 2018; Stanczyk and Clarke, 2010).

Contrary to our expectations, plasma cortisol in the present study was lower in the stress group than in the control, which suggests that UCS exposure resulted in downregulation of cortisol. Downregulation of the HPI axis is a common response during chronic stress and stress adaptation in several fish species (Barton, 2002; Madaro et al., 2015; Schreck and Tort, 2016; Xu et al., 2022). Similar to our study, wild-strain *S. salar* at juvenile stage exposed to repeated stressors for 42 days also showed lower plasma cortisol levels relative to the control group before and after acute stress (McCormick et al., 1998). However, the generally lower plasma cortisol of stressed fish found here is not consistent with the heightened or comparable plasma cortisol levels in domesticated *S. salar* exposed to repeated stressors or UCS relative to the undisturbed group reported by previous studies (Lai et al., 2021; Madaro et al., 2015, 2016), indicating possible strain-dependent influences. The discrepancy in the results could be further attributed to the differences in experimental design employed in the domesticated and wild studies. First, the plasma samples in stress-exposed fish were collected 1 h after a 5 min chasing period in related studies (Lai et al., 2021; Madaro et al., 2015, 2016), which may have caused acute spikes of cortisol, whereas no stressor was applied prior to sampling in this study. Moreover, the cortisol levels were measured at shorter intervals (every 1–7 days) and for shorter exposure periods (9–23 days) in those studies (Lai et al., 2021; Madaro et al., 2015, 2016), which made it possible to capture the initial fish stress responses. Cortisol levels in plasma tend to increase within minutes to a few days and may decrease through time, despite repeated stress application, as a result of desensitization, habituation, exhaustion of the endocrine system and increased metabolic clearance rate (Carbajal et al., 2019; Laberge et al., 2019; Lai et al., 2021; Madaro et al., 2015, 2016; Schreck and Tort, 2016). Lastly, stressors were applied at a higher frequency (2 or 3 times per day) in related studies (Lai et al., 2021; Madaro et al., 2015, 2016), which may have significant effects on the stress severity and consequently the physiological response and compensation of fish (Barton, 2002; Schreck and Tort, 2016). A lower frequency of stress episodes may impose milder stress severity and increase the time available for the fish to recover from the stressors (Schreck, 2000).

Given the relatively mild stress exposure compared with other studies, the reduced cortisol levels in the stress group can probably be explained by conditioning or habituation. After fish experience mild sequential stressors for a period of time, the magnitude and duration of the stress response can become attenuated as a result of physical and psychological conditioning during exposure to earlier stressors (Schreck and Tort, 2016). For example, random conditioning (where fish were subjected to random stressors once daily) and positive conditioning (where food was given to fish following brief and mild daily stressful experiences) reduced the cortisol response of wild-strain Chinook salmon (*Oncorhynchus tshawytscha*) to subsequent stressful events (Schreck et al., 1995). The UCS protocol employed in this study may have induced similar random conditioning and habituation effects on wild-strain *S. salar*, which helped them adapt better to the inherent stressful stimuli in captive conditions. However, intentional downregulation and habituation, which is generally beneficial, should not be confused with exhaustion, where stress overload causes a reduced capacity to physiologically respond to novel stressors (Schreck and Tort, 2016). Of note, domesticated *S. salar* showed a lower cortisol response when exposed to a novel stressor following 23 days of UCS exposure (with stress application 3 times per day; Madaro et al., 2015), indicating exhaustion. This might be the case for our wild-strain *S. salar*, especially as UCS induced growth reduction (discussed in ‘UCS-induced growth reduction and heart remodeling’, below), suggesting a state of chronic stress. Confirming the mechanism of cortisol attenuation requires further research where control and stress-exposed fish are exposed to novel stressors.

### Temporal profile of scale cortisol

Scale cortisol responded dynamically to changes in the plasma cortisol response over time. Scale cortisol level shows increasing promise as a biomarker of chronic stress as it indicates the cumulative activity of the physiological stress response over extended periods (Aerts et al., 2015; Laberge et al., 2019). To our knowledge, this study is the first to examine the temporal profile of scale cortisol in *S. salar.* Similar to studies that examined the scale cortisol accumulation in other fish species exposed to chronic stress (Aerts et al., 2015; Carbajal et al., 2018; 2019; Hanke et al., 2019, 2020; Laberge et al., 2019), a temporal increase in scale cortisol was also observed in this experiment. However, the cortisol accumulation in scales was only significant in control fish. The significant increase of scale cortisol in control despite the ‘stable’ plasma cortisol concentration in this treatment indicates the capacity of scales to incorporate circulating cortisol. A time-dependent increase of scale cortisol in the undisturbed (control) fish with a ‘stable’ plasma cortisol trend was also observed in an experiment with common dab (*Limanda limanda*; Vercauteren et al., 2022) and *O. mykiss* (Carbajal et al., 2019)*.*

Contrary to our expectations, the temporal incorporation of cortisol in the scales of stress fish was not significant and the scale cortisol in this treatment was generally lower compared with that in the control. Although inconsistent with the results of previous experiments in common carp (*Cyprinus carpio*; Aerts et al., 2015) and goldfish (*Carassius auratus*; Laberge et al., 2019) that showed higher cortisol accrual in the scales of fish exposed to UCS, the lower scale cortisol content in stressed fish here is intuitive given the UCS-induced downregulation of the cortisol response indicated by the plasma fluctuations noted in this study. Unlike the rapid downregulation of plasma cortisol levels, which was already apparent at week 2, however, the difference in scale cortisol levels between treatments was only significant at week 8. This indicates a delay in the incorporation of cortisol in the scales, which was also reported in *C. auratus* (Laberge et al., 2019) and *O. mykiss* (Carbajal et al., 2019). UCS hypothetically induced an increased plasma cortisol level in the first days of the exposure, resulting in a short-lived elevation in the scale cortisol (not captured because of the long sampling interval) that was eventually balanced out by the cortisol downregulation, hence the comparable scale cortisol level between treatments in the first weeks of this study.

### Cortisol regulation

#### Hypothalamus

The cortisol response exhibited by *S. salar* can be partly associated with the expression of genes involved in HPI regulation examined in this study. In the hypothalamus, *crh* was decreasingly expressed over time in both treatments, but the rate of decline was slower in the stress treatment and the overall expression of this gene was higher compared with control. Though the generally higher expression of *crh* in the stress-exposed group agrees with UCS studies on *S. salar* (Madaro et al., 2015), *C. carpio* (Aerts et al., 2015) and zebrafish (*Danio rerio*; Piato et al., 2011), this finding seems at odds with the generally lower cortisol response relative to the control. This may be due to the capacity of cortisol to elicit a direct negative feedback control on the *crh* expression (Bernier et al., 2004; Bernier and Peter, 2001). Moreover, *crh* expression does not necessarily correlate with (plasma) cortisol levels. For instance, UCS-exposed *S. salar* exhibited comparable plasma cortisol levels with control although *crh* was upregulated (Madaro et al., 2015). Also, how UCS-induced changes in gene expression, as measured by mRNA, represent the levels of proteins for which they encode was not explored in these studies and expression of HPI-related genes may not necessarily correlate with protein levels.

During chronic stress, the downregulation of the HPI axis in teleost fish is mediated by receptors involved in the negative feedback, GR and MR (Bury et al., 2003; Faught and Vijayan, 2018). Upregulation of GR and MR at key sites of the HPI axis has been associated with downregulated of the cortisol response of salmonids exposed to chronic stress (Kiilerich et al., 2018; Madaro et al., 2015). In this study, expression of *gr* in the hypothalamus was generally higher in the stress treatment, while *mr* expression was unaffected. The generally comparable expression of *mr* in the hypothalamus is in line with the results of related studies on salmonids (Kiilerich et al., 2018; Madaro et al., 2015, 2016) suggesting the minor role played by this receptor in HPI regulation at the hypothalamus level. However, the observed UCS-induced upregulation of *gr* expression in the hypothalamus disagrees with the unchanged or downregulated expression noted in salmonids exposed to UCS and a repeated stressor protocol for 7–23 days (Kiilerich et al., 2018; Madaro et al., 2015, 2016). Aside from the stress severity-dependent response of corticosteroid receptors (CRs) to chronic stress (Pavlidis et al., 2015), the discrepancy in the results can be attributed to the differences in the experimental period as the difference in *gr* expression between treatments was not significant until the 5th and 8th week of exposure in this study.

The expression of *11β-hsd2* was affected by time but not by treatment and their interaction: it was generally upregulated in both treatments at weeks 5 and 8. 11B-HSD2 is an enzyme involved in the inactivation of cortisol to cortisone, thereby making cortisol less available (Baker, 2004; Chapman et al., 2013). Although the lack of a treatment-related effect does not coincide with the UCS-induced downregulation of *11β-hsd2* documented for *S. salar* (Madaro et al., 2015), this result is interpreted together with the observed trend of other HPI mediators in an attempt to understand the cortisol response of *S. salar* in this study. Inhibition of 11β-HSD2 resulted in increased *crh* expression in *D. rerio* (Alderman and Vijayan, 2012), while *crh* abundance decreased in rainbow trout exposed to a GR antagonist (Alderman et al., 2012). As such, the temporal decline and the generally higher *crh* expression by UCS-exposed fish may have played a role in the time-dependent upregulation of *11β-hsd2* in both treatments coupled with the higher *gr* expression in the stress group.

#### Pituitary

The effect of UCS on the expression of genes involved in negative feedback control of cortisol in the pituitary was time dependent such that UCS-exposed fish seemed to exhibit higher expression of *gr*, *mr* and *11β-hsd2* at week 8. Similar to this, a stimulatory effect on the expression of these genes in *S. salar* following UCS was also reported and underlay the dampened cortisol response in these fish (Madaro et al., 2015). Considering this, responses of these genes probably played a role and supported the feedback systems in the hypothalamus in abating the cortisol levels of stressed fish and, thus, led to the lower scale cortisol levels observed.

Gene expression of *pomca1*, *pomca2* and *pomcb* paralogs that derived from the salmonid genome duplication (Kalananthan et al., 2020; Leder and Silverstein, 2006) analyzed in the pituitary showed time had significant effects on the expression of all analyzed *pomc* genes whilst treatment had a minimal effect on these genes. Among the POMC paralogs, only the expression of *pomca2* was significantly affected by treatment. However, this effect was mostly driven by the high pre-stress expression of this gene as *post hoc* analysis showed no difference between treatments from weeks 2 to 8. The generally non-significant impact of UCS on expression of *pomc* genes does not coincide with the upregulated expression reported in UCS studies on *S. salar* and *C. carpio* (Aerts et al., 2015; Madaro et al., 2015), which suggests that the response of POMC to UCS is protocol dependent. Of note, it was documented that *pomca1* and *pomcb* mRNA transcript abundance did not change following UCS exposure of *S. salar* (Madaro et al., 2015). The expression of *pomc* genes here seemed to generally increase from week 2 to 8 in both treatments, which was counterintuitive given the stable or declining plasma cortisol response in the control and stress groups, respectively. This suggests that other mechanisms probably induced a stronger influence on the cortisol response observed in this study. For instance, the time-dependent increase in *pomc* expression may have been overpowered by the decline in *crh* and UCS-induced upregulation of *gr*, *mr* and *11β-hsd2* in the hypothalamus and/or pituitary*.*

#### Head kidney

It was suggested that cortisol may exert an ultra-short negative feedback loop directly at the level of the head kidney (Samuel Bradford et al., 1992). Nevertheless, the expression of genes involved in cortisol release (*star*) and inactivation (*11β-hsd2*) was not affected by treatment, time and their interaction. Although the cortisol receptors were affected by time, treatment and interactive effects were not significant. Moreover, the effect of time was minimal: *post hoc* results showed that *gr* expression did not differ among treatment×time combinations and *mr* expression was generally comparable with pre-stress levels throughout the experiment. This finding suggests the hypothalamus and pituitary are the main sites of stress response modulation, which coincides with observations of previous studies (McEwen, 2006; Rotllant et al., 2000) including the UCS experiment on *S. salar* (Madaro et al., 2015).

### UCS-induced growth reduction and heart remodeling

Despite the downregulation of cortisol levels, UCS significantly reduced the growth performance of the fish. Madaro et al. (2015) reported a 41.0% growth reduction in UCS-exposed *S. salar* and this was attributed to the appetite-suppressing effects of both CRH and cortisol. Although the effects of UCS on feed intake were not examined in this study, *crh* expression levels were generally higher in the stress treatment and may have induced similar appetite suppression and growth reduction to that noted in previous studies (Madaro et al., 2015; Bernier and Peter, 2001; Ortega et al., 2013). However, the observed UCS-induced growth rate reduction was counterintuitive given the lower cortisol levels in the stress treatment. Nevertheless, chronic stress studies documented inconsistency between cortisol levels and growth in salmonids and other fish species, suggesting that growth suppression induced by chronic stressors is likely to be mediated by other factors aside from GCs (Madaro et al., 2015; 2016; Van Weerd and Komen, 1998). For instance, reduced feed intake and conversion efficiency have been associated with growth repression in chronic stress-exposed salmonids (Madaro et al., 2015; Pickering and Stewart, 1984) and rare minnow (*Gobiocypris rarus*; Xu et al., 2022) with a downregulated cortisol response. Moreover, chronic stress exposure has been found to directly affect the growth hormone and insulin-like growth factor (GH/IGF) system, which is the main promoter of muscle growth in fish. In the study of Valenzuela et al. (2018) on fine flounder (*Paralichthys adspersus*), cortisol downregulation to control levels and growth reduction were observed following a 7 week crowding exposure, and the negative effect on growth was primarily attributed to the downregulation of the GH/IGF system directly imposed by the stress exposure (Valenzuela et al., 2018).

Previous studies showed that cortisol administration and high cortisol responsiveness promoted heart enlargement and remodeling in *O. mykiss* and *S. trutta* (Johansen et al., 2011, 2017; Norstrud et al., 2018). Cardiac growth and changes in compact myocardium have also been observed in salmonids during stressful circumstances including spawning migration and thermal acclimation (Franklin and Davie, 1992; Gamperl and Farrell, 2004). Cortisol's involvement in cardiac enlargement seems logical given the noted increases in plasma cortisol levels during these periods (Carruth et al., 2000; Tromp et al., 2018). To the best of our knowledge, this is the first study to elucidate the effect of UCS on cardiac remodeling in *S. salar*. Here, the compact myocardium width, compact/spongy myocardium area and extra-bundular sinus area of undisturbed and UCS-exposed fish were comparable. However, the width and proportion of the compact myocardium had a weak yet significantly linear correlation with scale cortisol, which could be related to the cortisol responsiveness of individuals (Johansen et al., 2011). Interestingly, the linear correlation between cortisol and compact myocardium of the individuals was only significant with scale cortisol but not with plasma cortisol levels. It was documented that individual cortisol responsiveness in *O. mykiss* is associated with compact myocardium area, where individuals that responded with higher plasma cortisol levels following acute stress developed thicker compact myocardium than those with low cortisol responses (Johansen et al., 2011). However, basal plasma cortisol levels, which were determined in this study, do not necessarily reflect individual cortisol responsiveness (Ferrari et al., 2020), and may therefore exhibit poor correlations with cardiac morphology. In contrast, high cortisol responsive individuals exposed to a repeated chronic stress protocol tend to accumulate higher cortisol levels in scales than low responders (Samaras et al., 2021). Beside this, morphological remodeling of the heart is generally a long-term process driven by prolonged factors and would probably correlate better with a more conserved biomarker.

UCS-exposed fish exhibited generally higher RVM relative to the control but the role played by cortisol seems trivial given the downregulated cortisol response of these fish. Of note, the increase in RVM may be driven by the UCS-induced reduction in BM, which seemed to explain the reduction of RVM at weeks 2–8 in the control relative to the pre-stress condition. Unlike previous studies with cortisol-fed salmonids (Johansen et al., 2011; 2017), an increase in absolute ventricular mass was not observed in this study. Similarly, a non-significant increase in absolute ventricular mass was also observed in cortisol-exposed *O. mykiss* and the rise in RVM was attributed to the cortisol-induced growth reduction (Nørstrud et al., 2018).

Nevertheless, the observed lack of correlation between endogenous cortisol and RVM suggests that other mechanisms may be involved. It was documented that isolated *O. mykiss* showed a time-dependent increase in RVM even though cortisol remained at basal levels throughout the experiment (Norstrud et al., 2018). Though the basal cortisol levels and increased feed intake suggest that isolation was not stressful for the fish, it was argued that other (unquantified) stress hormones could be elevated by stressful stimuli such as social isolation and consequently contribute to the increased RVM (Norstrud et al., 2018). As discussed by Norstrud et al. (2018), catecholamines and monoamine serotonin are known to be stimulated by stress and were documented to induce cardiac remodeling in mammals (Lairez et al., 2013; Zimmer, 2003). While catecholamines showed slight effects on the myocardium in fish (Tota et al., 2010), the known hypertrophy-inducing capacity of monoamine serotonin in mammals has not been confirmed in fish. Aside from these, elevated levels of androgen (testosterone, 11-ketotestosterone) have been identified as the primary stimulant of cardiac growth in salmonids during spawning (Gamperl and Farrell, 2004).

How these observed morphological changes in the ventricle affect the cardiac performance of the fish is a question that will be answered in our upcoming experiments. Plasticity in size, geometry and myocardial proportion of the ventricle can be adaptive responses to maintain or improve the cardiac performance in salmonids exposed to stressful circumstances (Gamperl and Farrell, 2004). Bigger hearts and higher RVM were associated with increased stroke volume, and adjustments in myocardium layers (compact versus spongy muscle) affect the force of contraction (Gamperl and Farrell, 2004). Both types of morphological remodeling may contribute to cardiac output enhancement, which supports the increased functional demands placed on the heart of salmonids during challenging periods, including thermal acclimation and spawning migration (Gamperl and Farrell, 2004; Keen et al., 2017). Nevertheless, the cortisol-induced heart enlargement and thickening of the compact myocardium in salmonids observed by Johansen et al. (2017) corresponded with impaired cardiovascular performance, indicating maladaptive effects of cortisol. Moreover, farming aquaculture techniques seem to induce maladaptive cardiac remodeling and the differences in stress levels experienced by the fish through time seem to be the underlying factor in this remodeling (Frisk et al., 2020). It was reported that the ventricular characteristics observed in a more intensive, growth-promoting (at least during juvenile stages) aquaculture techniques resembled that of cortisol-induced ventricular remodeling (i.e. ventricle enlargement and higher compact myocardium thickness; Johansen et al., 2011, 2017) and presented a higher risk for cardiac rupture and mortality (Frisk et al., 2020). To explain the discrepancy in cardiac stress responses, Johansen et al. (2017) proposed that the cortisol-stimulated hypertrophy should be accompanied by other factors (i.e. anabolic sex steroids during spawning migration) to elicit adaptive functional changes in the heart. Given the divergence in the stress-related effects on fish cardiac performance, it is difficult to ascertain whether the observed morphological changes following UCS are adaptive or maladaptive without further research.

### Conclusion

Unlike the widely explored acute stress response of fish, information on the effects of chronic stress remains relatively limited. Accurate understanding of the chronic stress response is further complicated by the use of stress biomarkers that fail to reflect the stress status over extended periods (i.e. plasma cortisol) in previous studies. This study quantified the temporal profile of cortisol in plasma and scales and explored the mechanisms that regulate the HPI axis of wild-strain *S. salar* juvenile exposed to UCS for 8 weeks. The correlation of quantified stress indicators was further established with growth and cardiac morphology. Our results demonstrated the suitability and applicability of scale cortisol to reflect chronic cortisol elevation over time, as indicated by the temporal accumulation of cortisol in scales, which corresponded with the ‘snap-shot’ plasma levels. The UCS protocol employed in this study resulted in cortisol downregulation below control levels. The growth reduction induced by UCS indicates that this downregulation is probably due to the exhaustion of the HPI axis instead of conditioning or habituation, but further studies are required to confirm this. Corresponding with the UCS-induced downregulation of cortisol, a general upregulation of stress axis genes involved in the inactivation and negative feedback of cortisol at the hypothalamus and pituitary level was observed. When associated with organismal performance, the downregulated cortisol levels did not correlate with the growth suppression induced by UCS. However, the observed linear correlation of compact myocardium with scale cortisol levels, but not with plasma cortisol levels, suggests the involvement of ‘chronic’ cortisol in cardiac remodeling and highlights the importance of a retrospective stress biomarker, for which scale cortisol is showing great potential when associating chronic stress with long-term processes such as cardiac remodeling.

## Supplementary Material

10.1242/jexbio.246504_sup1 Supplementary information
