# Supplementary material for: Long-term stress induced cortisol downregulation, growth reduction and cardiac remodeling in Atlantic salmon
Source: J Exp Biol. 2023 Nov 16;226(22):jeb246504. doi: 10.1242/jeb.246504 (PMC10690108; doi:10.1242/jeb.246504)
Supplement: Supplementary information [file jexbio-226-246504-s1.pdf]

Table S1. Description of how the acute stressors were applied.

Type of stressor	Stress duration or intensity	Procedure
Chasing	5-10 minutes	Chase fish in tank with a net to induce escape response.
Netting + Air Exposure	3-7 minutes	Lower the water level (40-50%) and net the fish out of the water at random. Expose netted fish to air for 5-30 seconds.
Netting + Crowding	3-6 minutes	Lower the water level until the dorsal fin is exposed (95%). Chase the fish in tank.
Temperature Shock	+1-3°C; 1-2 hours	After air exposure and crowding stress, refill the tank with warmer water (up to 3°C difference) and allow the thermal regulator to cool the water down to set temperature (13± 0.5°C).

Table S2. Randomized acute stressors applied daily during the experiment.

Sampling	Date	Day	Time of application (± 00:30)	Type of stressor	Duration (min)	Temperature shock (± 0.5°C)
Week 0	31-May	Tuesday	N/A	N/A	N/A	N/A
	01-Jun	Wednesday	10:00	Netting+Air Exposure	4 minutes	+1.5°C
	02-Jun	Thursday	14:00	Netting+Crowding	3 minutes	+2°C
	03-Jun	Friday	17:00	Netting+Air Exposure	7 minutes	+1°C
	04-Jun	Saturday	11:00	Netting +Crowding	5 minutes	+1°C
	05-Jun	Sunday	13:00	Chasing	10 minutes	N/A
	06-Jun	Monday	11:00	Netting+Air Exposure	6 minutes	+1.5°C
	07-Jun	Tuesday	9:00	Netting+Crowding	5 minutes	+2.5°C
	08-Jun	Wednesday	13:00	Netting+Air Exposure	7 minutes	+1°C
	09-Jun	Thursday	15:00	Chasing	8 minutes	N/A

Journal of Experimental Biology • Supplementary information

Table S2 (continued)

Sampling	Date	Day	Time of application (± 00:30)	Type of stressor	Duration (min)	Temperature shock (± 0.5°C)
	10-Jun	Friday	16:00	Netting+Air Exposure	4 minutes	+2.5°C
	11-Jun	Saturday	14:00	Netting+Crowding	3 minutes	+1°C
	12-Jun	Sunday	12:00	Chasing	7 minutes	+2°C
	13-Jun	Monday	8:00	Netting+Air Exposure	5 minutes	+1.5°C
Week 2	14-Jun	Tuesday	N/A	N/A	N/A	N/A
	15-Jun	Wednesday	12:00	Chasing	8 minutes	N/A
	16-Jun	Thursday	14:00	Netting+Air Exposure	6 minutes	+2°C
	17-Jun	Friday	12:00	Chasing	10 minutes	N/A
	18-Jun	Saturday	16:00	Chasing	10 minutes	N/A
	19-Jun	Sunday	14:00	Chasing	8 minutes	N/A
	20-Jun	Monday	11:00	Netting+Air Exposure	5 minutes	+1°C
	21-Jun	Tuesday	10:00	Netting+Crowding	4 minutes	+2°C
	22-Jun	Wednesday	16:00	Chasing	10 minutes	N/A
	23-Jun	Thursday	9:00	Chasing	10 minutes	N/A
	24-Jun	Friday	16:00	Netting+Crowding	5 minutes	+1.5°C
	25-Jun	Saturday	13:00	Netting+Crowding	3 minutes	+2°C
	26-Jun	Sunday	16:00	Chasing	8 minutes	N/A
	27-Jun	Monday	10:00	Netting+Air Exposure	3 minutes	+1°C
	28-Jun	Tuesday	14:00	Chasing	7 minutes	N/A
	29-Jun	Wednesday	13:00	Chasing	8 minutes	N/A

Table S2 (continued)

Sampling	Date	Day	Time of application (± 00:30)	Type of stressor	Duration (min)	Temperature shock (± 0.5°C)
	30-Jun	Thursday	11:00	Netting+Crowding	4 minutes	+3°C
	01-Jul	Friday	10:00	Netting+Crowding	5 minutes	+2°C
	02-Jul	Saturday	16:00	Chasing	6 minutes	N/A
	03-Jul	Sunday	12:00	Netting+Crowding	3 minutes	+1°C
	04-Jul	Monday	13:00	Netting+Air Exposure	3 minutes	+1°C
Week 5	05-Jul	Tuesday	N/A	N/A	N/A	N/A
	06-Jul	Wednesday	10:00	Chasing	10 minutes	N/A
	07-Jul	Thursday	14:00	Chasing	8 minutes	N/A
	08-Jul	Friday	11:00	Netting+Crowding	5 minutes	+2°C
	09-Jul	Saturday	8:00	Netting+Crowding	6 minutes	+1°C
	10-Jul	Sunday	13:00	Netting+Air Exposure	4 minutes	+1.5°C
	11-Jul	Monday	12:00	Netting+Crowding	5 minutes	+2.5°C
	12-Jul	Tuesday	11:00	Chasing	10 minutes	N/A
	13-Jul	Wednesday	10:00	Netting+Crowding	6 minutes	+3°C
	14-Jul	Thursday	8:00	Netting+Air Exposure	5 minutes	+1°C
	15-Jul	Friday	15:00	Chasing	5 minutes	N/A
	16-Jul	Saturday	10:00	Chasing	5 minutes	N/A
	17-Jul	Sunday	16:00	Netting+Crowding	3 minutes	+1.5°C
	18-Jul	Monday	9:00	Netting+Air Exposure	4 minutes	+2°C
	19-Jul	Tuesday	17:00	Chasing	5 minutes	N/A

Table S2 (continued)

**Sampling**	**Date**	**Day**	**Time of application (± 00:30)**	**Type of stressor**	**Duration (min)**	**Temperature shock (± 0.5°C)**
	20-Jul	Wednesday	11:00	Chasing	10 minutes	N/A
	21-Jul	Thursday	10:00	Netting+Crowding	5 minutes	+1°C
	22-Jul	Friday	9:00	Netting+Crowding	6 minutes	+3°C
	23-Jul	Saturday	12:00	Netting+Air Exposure	7 minutes	+2.5°C
	24-Jul	Sunday	15:00	Netting+Air Exposure	6 minutes	+1.5°C
	25-Jul	Monday	12:00	Chasing	9 minutes	N/A
Week 8	26-Jul	Tuesday	N/A	N/A	N/A	N/A
